# Mortality in members of HIV-1 serodiscordant couples in Africa and implications for antiretroviral therapy initiation: Results of analyses from a multicenter randomized trial

**DOI:** 10.1186/1471-2334-12-277

**Published:** 2012-10-30

**Authors:** Guy de Bruyn, Amalia Magaret, Jared M Baeten, Jairam R Lingappa, Patrick Ndase, Connie Celum, Anna Wald

**Affiliations:** 1 Perinatal HIV Research Unit, University of the Witwatersrand, Johannesburg, South Africa; 2 Departments of Laboratory Medicine, University of Washington, Seattle, WA, USA; 3 Departments of Epidemiology, University of Washington, Seattle, WA, USA; 4 Departments of Global Health, University of Washington, Seattle, WA, USA; 5 Departments of Medicine, University of Washington, Seattle, WA, USA; 6 Departments of Paediatrics, University of Washington, Seattle, WA, USA; 7 Vaccine and Infectious Diseases Division, Fred Hutchinson Cancer Research Center, Seattle, WA, USA; 8 Present address: Sanofi Pasteur, Discovery Drive, Swiftwater, PA, USA

## Abstract

**Background:**

The risk of HIV-1 related mortality is strongly related to CD4 count. Guidance on optimal timing for initiation of antiretroviral therapy (ART) is still evolving, but the contribution of HIV-1 infection to excess mortality at CD4 cell counts above thresholds for HIV-1 treatment has not been fully described, especially in resource-poor settings. To compare mortality among HIV-1 infected and uninfected members of HIV-1 serodiscordant couples followed for up to 24 months, we conducted a secondary data analysis examining mortality among HIV-1 serodiscordant couples participating in a multicenter, randomized controlled trial at 14 sites in seven sub-Saharan African countries.

**Methods:**

Predictors of death were examined using Cox regression and excess mortality by CD4 count and plasma HIV-1 RNA was computed using Poisson regression for correlated data.

**Results:**

Among 3295 HIV serodiscordant couples, we observed 109 deaths from any cause (74 deaths among HIV-1 infected and 25 among HIV-1 uninfected persons). Among HIV-1 infected persons, the risk of death increased with lower CD4 count and higher plasma viral levels. HIV-1 infected persons had excess mortality due to medical causes of 15.2 deaths/1000 person years at CD4 counts of 250 – 349 cells/μl and 8.9 deaths at CD4 counts of 350 – 499 cells/μl. Above a CD4 count of 500 cells/μl, mortality was comparable among HIV-1 infected and uninfected persons.

**Conclusions:**

Among African serodiscordant couples, there is a high rate of mortality attributable to HIV-1 infection at CD4 counts above the current threshold (200 – 350 cells/μl) for ART initiation in many African countries. These data indicate that earlier initiation of treatment is likely to provide clinical benefit if further expansion of ART access can be achieved.

**Trial Registration:**

Clinicaltrials.gov (NCT00194519)

## Background

HIV-1-related mortality increases as the CD4 count in peripheral blood decreases [1]. Initiation of antiretroviral therapy (ART) at a CD4 count above 200 cells/μl reduces mortality of HIV-1 infected persons compared to therapy that is delayed until CD4 count declines below 200 cells/μl [2], and adopting an even higher CD4 threshold for initiating ART may further reduce mortality [3-5]. Recently, evidence from a clinical trial demonstrated that initiation of ART at a CD4 count between 350 and 550 cells/μl results in clinical benefits and reduces transmission to HIV-uninfected partners [6]. World Health Organization (WHO) guidelines recommend that therapy be initiated when an HIV-1 infected person’s CD4 count falls to 350 cells/μl [7]. Currently, the CD4 threshold is 200 –250 cells/μl in most African countries, and 350 cells/μl in others, where the WHO guidelines have been adopted.

However, controversy remains regarding the ideal CD4 threshold at which ART should be initiated [3-5,8], and data to inform guidelines are still limited, especially from resource-poor settings. A large proportion of excess adult mortality in sub-Saharan Africa is HIV-1-related [9]. Excess HIV-1-related mortality has been shown to be related to several clinical factors, such as use of ART, CD4 count, clinical stage of disease, as well as demographic factors, including migration, level of educational attainment, age, and economic status [9-15]. Differential HIV-related mortality, based on access to care and socioeconomic status, has been observed in developed countries [16]. The impact of these factors may be accentuated in developing countries, where access to health care is more limited in general.

We compared mortality among HIV-1 infected and uninfected partners in African HIV-1 serodiscordant couples participating in the Partners in Prevention HSV/HIV Transmission Study, a clinical trial of acyclovir for HSV-2 suppression among HSV-2/HIV-1 dually-infected persons for prevention of HIV-1 transmission to their HIV-1 uninfected partners. As reported previously, acyclovir suppression did not reduce HIV-1 transmission to uninfected partners in spite of a 0.25 log_10_ reduction in plasma HIV-1 levels [17], but modestly delayed progression in HIV-1 infected persons [18]. Because most participating couples were cohabiting, the HIV-1 uninfected partners comprised an excellent control group for determining mortality attributable to HIV-1 infection, while accounting for shared exposures such as a common environment, socioeconomic status, diet, exposure to infectious diseases, and access to care.

## Methods

### Study population and procedures

Between November 2004 and April 2007, 3408 HIV-1 serodiscordant couples in 7 African countries (Botswana, Kenya, Rwanda, South Africa, Tanzania, Uganda, and Zambia) were enrolled into a randomized, double-blind, placebo-controlled trial of twice daily 400 mg acyclovir in HIV-1/HSV-2 dually infected partners to prevent HIV-1 transmission to the uninfected partner. HIV-1-infected partners were required to be 18 years of age or older, seropositive for HIV-1 and HSV-2, and have a CD4 count ≥250 cells/μl. Persons who had a prior AIDS-defining diagnosis, reported taking ART, had prior adverse reactions to acyclovir or planned use of anti-HSV antivirals, or were pregnant were excluded. For HIV-1 infected participants, CD4 counts were measured at enrolment and 6 month intervals and plasma viral load at enrolment, 3, 6, 12 months, and study exit. Those meeting national criteria for ART initiation during follow-up were offered ART through referral to local clinics or at the study site. HIV-1-infected participants were followed monthly for up to 24 months after enrolment, and HIV-1-uninfected partners were seen quarterly. Study procedures have been detailed elsewhere [17-20].

### Medical monitoring and assignment of causes of death

For participants who died during follow-up, information on the putative cause of death was obtained from the surviving partner, family members, and/or medical records, if available. The cause of death was classified as a medical death or traumatic death, such as traffic collisions or assault. The study medical monitor (AW) assigned the medical cause of death to one of the following broad categories based on information provided by the local site: pneumonia or other respiratory illness; malaria; tuberculosis; gastrointestinal infections and other disorders; other infections; or other medical causes.

### Laboratory analyses

HIV-1 serostatus was determined by dual rapid tests with confirmatory EIA and HSV-2 serostatus by HerpeSelect-2 EIA (Focus Technologies, Cypress, CA). HIV-1 and HSV-2 serostatus were confirmed in batch by the University of Washington Western blot and only those who were confirmed were included in the analysis [19]. CD4 testing was performed at study sites using standard flow cytometry (BD Biosciences, San Jose, CA), under an external quality assurance program. Plasma HIV-1 RNA was quantified in acid citrate dextrose (ACD) using the 96-test COBAS AmpliPrep/COBAS TaqMan HIV-1 RNA assay, version 1.0 (Roche Diagnostics, Indianapolis, IN) at the University of Washington, with a limit of quantification of 240 copies/mL.

### Ethical review

The University of Washington Human Subjects Review Committee and ethical review committees at each local and collaborating institution approved the study protocol. All participants provided written informed consent.

### Statistical analyses

Data were analyzed using SAS (version 9.2, SAS, Cary, NC). Incidence rates were computed by summing the time at risk within each range of predictors. Participants contributed follow-up time to more than one category of CD4 count, plasma HIV-1 RNA and WHO HIV stage, as their condition changed over time. Participants who were HIV-1 infected at enrolment were analyzed separately from their partners to examine potential time-varying predictors of death. Follow-up time for HIV-1 seronegative partners was censored at HIV-1 seroconversion. We compared the proportion dying while on study within couples using McNemar’s test. Paired Cox regression was used to examine baseline predictors of death within couples. Ties were handled using the Efron method and each analysis was stratified by site. Final, multivariate models were determined using backward selection from all predictors significant in univariate analysis at the p < 0.1 level. Because of strong associations with CD4 and HIV viral load, WHO HIV stage was excluded from multivariate analysis. Treatment arm (acyclovir versus placebo) and gender were retained to determine associations after adjustment for other predictors included in the final model. Of note, ART use did not include antiretrovirals used for prevention of mother-to-child transmission of HIV-1.

To evaluation simultaneously the influence of HIV-1 status and, among those HIV-1-seropositive, time-varying measures of immune suppression, generalized estimating equations (GEE) were performed with a Poisson link, an exchangeable correlation structure, and log(time) as an offset [21]. This method took advantage of the paired nature of study participants and allowed appropriate handling of the association between couples, while also permitting generalization of subsequent inference to between-person differences in HIV-1 status. The Poisson link in these GEE models provided risk ratios for the incidence of death, and permitted computation of excess mortality attributable to HIV-1 via transformation of regression coefficients. The delta-method was used to compute the variability of excess mortality (see Additional file 1: Methods) [22]. Our primary analyses examined the risk for death from a medical cause; to assess the robustness of our findings, sensitivity analyses were performed.

## Results

We included in the analysis the 3295 couples whose HIV-1 and HSV-2 status were confirmed and for whom both partners had at least one follow-up visit (Table 1). Median follow-up time was 20 months among HIV-1-infected partners, 18 months among HIV-1-uninfected partners and 20 months overall (interquartile range (IQR), 15 – 24). Retention among HIV-1-infected partners was 97% at 12 months (2999 expected visits) and 92% at 24 months (of 1348 visits). Retention among HIV-uninfected partners was 94% at 12 months (of 3107 expected visits) and 85% at 24 months (of 1449 expected visits).

**Table 1:** Demographic and clinical characteristics, by partner type

**Characteristic**	**HIV-1 infected partner**		**HIV-1-uninfected partner**	
	**(N = 3295)**		**(N = 3295)**	
	**Men**	**Women**	**Men**	**Women**
	**(n = 1073)**	**(n = 2222)**	**(n = 2222)**	**(n = 1073)**
Age, median (IQR)	37 (32,45)	30 (25, 35)	35 (30, 42)	31 (25, 38)
Informal settlement^¶^	404 (38%)	859 (39%)	869 (39%)	390 (39%)
Married to study partner	866 (81%)	1627 (73%)	1636 (74%)	864 (81%)
Cohabiting with study partner	1002 (93%)	1970 (89%)	1983 (89%)	1000 (93%)
>8 years of education completed	512 (48%)	901 (41%)	1157 (52%)	401 (37%)
Any income	638 (59%)	546 (25%)	1331 (60%)	280 (26%)
CD4, median, cells/mm^3^ (IQR)			-	-
Enrollment	426 (334, 571)	481 (354, 664)		
Final visit	394 (276, 544)	437 (312, 617)		
HIV-1 plasma RNA, median, log_10_ copies/mL (IQR)			-	-
Enrollment	4.2 (3.5, 4.8)	3.8 (3.0, 4.4)		
Final visit	4.4 (3.6, 5.0)	4.0 (3.2, 4.7)		
Ever on ART over follow up	112 (10%)	178 (8%)	-	-
Highest WHO stage of HIV-1			-	-
< 3	806 (75%)	1720 (77%)		
3	243 (23%)	436 (20%)		
4	24 (2%)	66 (3%)		

¶Informal Settlement, as defined by study participants.

**Figure 1:**
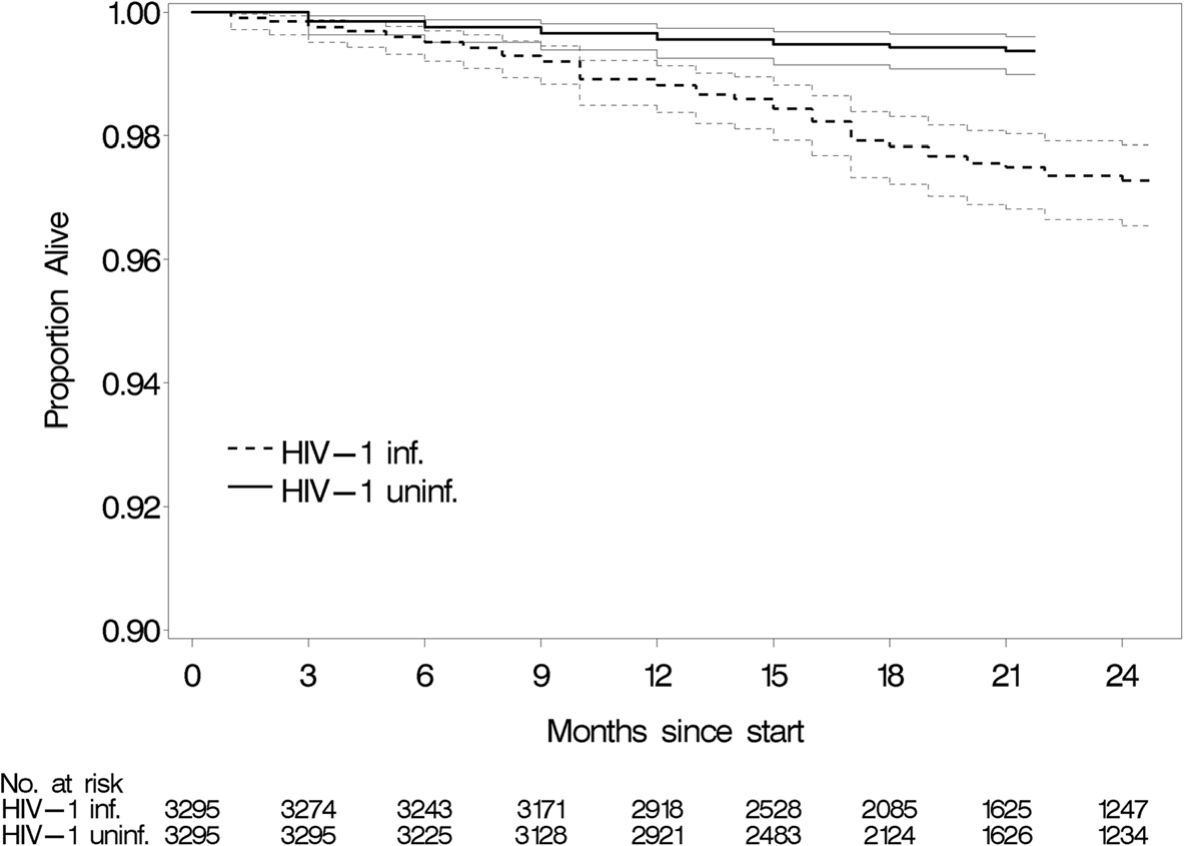
**Kaplan-Meier curve showing mortality with 95% CI caused by medical illness by participant serostatus**.

Among these couples, 74 HIV-1 infected participants and 25 HIV-1 uninfected participants died of any cause over the course of follow-up. In one couple, both the HIV-1 infected and HIV-1 uninfected partners died (from two differing medical causes). Trauma was the cause of death in three HIV-1 infected participants and in seven HIV-1 uninfected participants. Thus, 71 deaths among the HIV-1 infected participants and 18 among the HIV-1 uninfected participants were caused by a medical illness (Figure 1).

### Mortality among HIV-1 infected participants

First we performed Cox regression to examine the risk factors for death among HIV-1 seropositive participants. In univariate Cox regression, lower CD4 count, higher plasma HIV-1 RNA, and more advanced WHO stage of HIV-1 disease were significant predictors of medical deaths (Table 2). In a multivariate Cox model, CD4 count, plasma viral load (PVL), and ART use remained significant as predictors of death cause by medical illness.

**Table 2:** Univariate and multivariate Cox regression models of predictors of death attributed to medical causes among HIV-1 infected participants

	**Events**	**P-Y**	**Incidence**	**HR (95%CI)**	***P***	**aHR (95%CI)**	***P***
**CD4 cell count**							
>500	10	2062	4.8	ref		Ref	
350-499	20	1532	13.1	2.7 (1.2, 5.6)	.013	2.2 (1.0, 4.9)	.053
250-349	23	1154	19.9	4.1 (1.9, 8.6)	<.001	2.6 (1.2, 5.9)	.018
<250	18	422	42.7	8.0 (3.6, 17.7)	<.001	3.5 (1.4, 8.5)	.005
**Plasma HIV-1 RNA**							
<10 K	19	2465	7.7	ref		Ref	
10 K-50 K	5	1340	3.7	0.5 (0.2, 1.2)	.12	0.5 (0.2, 1.3)	.15
50 K-100 K	7	503	13.9	1.7 (0.7, 4.1)	.21	1.4 (0.6, 3.6)	.42
>100 K	40	853	46.9	5.7 (3.2, 10.0)	<.001	†5.8 (2.9, 11.4)	<.001
**Antiretroviral use**							
No	60	4973	12.1	ref		ref	
Yes	11	197	55.8	5.6 (2.7, 11.5)	<.001	†9.8 (4.1, 23.8)	.001
**WHO HIV Stage**							
1-2	36	4143	8.7	Ref		ND	
3	24	988	24.3	3.1 (1.8, 5.1)	<.001		
4	11	39	282.1	15.7 (6.3, 39.4)	<.001		
**Gender**							
Men	28	1708	16.4	Ref		Ref	
Women	43	3462	12.4	0.7 (0.4, 1.1)	.12	0.9 (0.6, 1.5)	.79
**Age**							
18-25	8	736	10.9	Ref		ND	
26-35	29	2417	12.0	1.2 (0.5, 2.6)	.67		
36+	34	2015	16.9	1.7 (0.8, 3.6)	.21		
**Type of housing**							
Formal	38	3050	12.5	ref		ND	
Informal	32	2001	16.0	1.6 (1.0, 2.7)	.071		
**Arm**							
Placebo	38	2562	14.8	Ref		ref	
Acyclovir	33	2607	12.7	0.8 (0.5, 1.3)	.44	1.0 (0.6, 1.6)	.99

*HR* hazard ratio, *N* number of deaths, *ND* not done, *NS* non-significant in the multivariate model, *P-Y* person-years. Covariates CD4 count, VL, Antiretroviral use, and WHO stage were all time-varying.

† An interaction term was also present in the model between high VL and ART (p = .004), demonstrating that the hazard of death associated with HIV viral load >100 K is no greater than with viral load <10 K for those on ART (p = .42).

Two hundred and ninety persons initiated ART. ART use accounted for 3.8% of follow-up time of HIV-1 seropositive partners (197 of 5170 person-years). Median CD4 count when last measured in clinic prior to ART initiation was 221 (range 11 – 1618). Median time between most recent CD4 count measurement at clinic and first report of ART initiation was 4 months (range 0 – 10). Eleven persons died following ART initiation: six died within three months of ART initiation and an additional four persons died between four and six months after initiation. The crude incidence rate of death prior to ART use was 63 per 4972 person year,s or 13 per 1000, while the incidence following ART initiation was 11 in 197 person years, or 56 per 1000. These rates can be further broken down into 6 in 69 person years (87 per 1000) within six months of ART initiation and 5 in 128 person-years (39 per 1000) at least six months after initiation.

### Comparison of mortality within couples

The number of deaths from medical causes was higher among HIV-1 infected participants than HIV-1 uninfected participants (71 vs. 18 deaths, p <0.001, respectively). In sensitivity analyses, these differences remained significant when we restricted the analysis to married couples who reported living together (55 vs. 14, p < 0.001), or to deaths that occurred within the first year of follow up (38 vs. 14, p = 0.001), or all-cause mortality, including trauma (74 vs. 25, p < 0.001).

Using paired Cox regression (Table 3), we found that the rate of death caused by a medical illness was higher in HIV-1 infected participants compared with HIV-1 uninfected participants, 13.7 vs. 3.6 deaths/1000 person-years (Hazard Ratio [HR] 3.7, 95% confidence interval [CI]: 2.2 – 6.2, p < 0.001). The effect of gender on mortality was confounded by HIV serostatus. Men and women showed similar unadjusted medical mortality (8.5 vs. 9.0 deaths/1000 person years; HR 1.0, 95%CI: 0.7 – 1.6, p = 0.86). Among HIV-1 uninfected participants, women showed lower all-cause mortality rates than men (p = 0.041), and a similar trend was seen in HIV-1-infected participants (p = 0.084), but no statistically significant differences were seen in death rates due to medical causes. The ten non-medical deaths observed occurred in one woman and nine men. No age effects were noted (data not shown).

**Table 3:** Comparison of death rates by partner type and gender

**Stratum**	**Covariate**	**Deaths**	**P-Y**	**Mortality per 1000 PY**	**Hazard Ratio (95%CI)**	**p-value**
Medical Deaths						
Included couples	HIV-1 uninfected	18	5009	3.6	Ref	<.001
	HIV-1 infected	71	5170	13.7	3.7 (2.2, 6.2)	
	Men	43	5059	8.5	Ref	.86
	Women	46	5120	9.0	1.0 (0.7, 1.6)	
HIV-1 uninfected	Men	15	3351	4.5	Ref	.12
	Women	3	1658	1.8	0.4 (0.1, 1.3)	
HIV-1 infected	Men	28	1708	16.4	Ref	.12
	Women	43	3462	12.4	0.7 (0.4, 1.1)	
All Deaths						
Included couples	HIV-1 uninfected	25	5009	5.0	Ref	<.001
	HIV-1 infected	74	5170	14.3	2.8 (1.8, 4.4)	
	Men	52	5059	10.2	Ref	.53
	Women	47	5120	9.2	0.9 (0.6, 1.3)	
HIV-1 uninfected	Men	22	3351	6.6	Ref	.041
	Women	3	1658	1.8	0.3 (0.1, 0.9)	
HIV-1 infected	Men	30	1708	17.6	Ref	
	Women	44	3462	12.7	0.7 (0.4, 1.0)	

*PY* person years.

### Causes of death

In most cases, limited data were available to further characterize the cause of death (Table 4). The largest number of deaths was categorized as ‘Other, medical’. Many of these deaths may have been caused by opportunistic infections, but limited clinical information from persons who did not receive care, or inability to obtain medical records in other cases, did not permit specific diagnoses; examples of narratives provided by the study sites are shown in the footnote to Table 4.

**Table 4:** Causes of death by partner type

	**HIV-1 infected (%)**	**HIV-1 uninfected (%)**
Trauma or Injury	3 (4%)	7 (28%)
Pneumonia and other respiratory illness	14 (19%)	1 (4%)
Malaria	5 (7%)	0 (0%)
Tubercμlosis	12 (16%)	4 (16%)
Gastrointestinal infections and related disorders	9 (12%)	1 (4%)
Other infections	6 (8%)	1 (4%)
Other medical^¶^	25 (34%)	11 (44%)
All Deaths	74 (100%)	25 (100%)

¶ “Other medical” conditions included any other conditions that coμld not be explicitly ascribed to the other categories of medical illness based on the available data, and did not resμlt from trauma. Examples of causes of death that were included in the ‘other medical’ category include the following site reports: 1) “Death following a short illness. Was asthmatic”; 2) “Death following severe headache.” 3) “Participant hospitalized with abdominal pains, headache, and joint pains. Died on the same day”. 4) “Sharp left sided chest pain and vomiting for 2 days, leading to death.” 5) “Death after admission due to gastroenteritis and hepatic encephalopathy, died on day of admission.”

### Excess mortality

**Figure 2:**
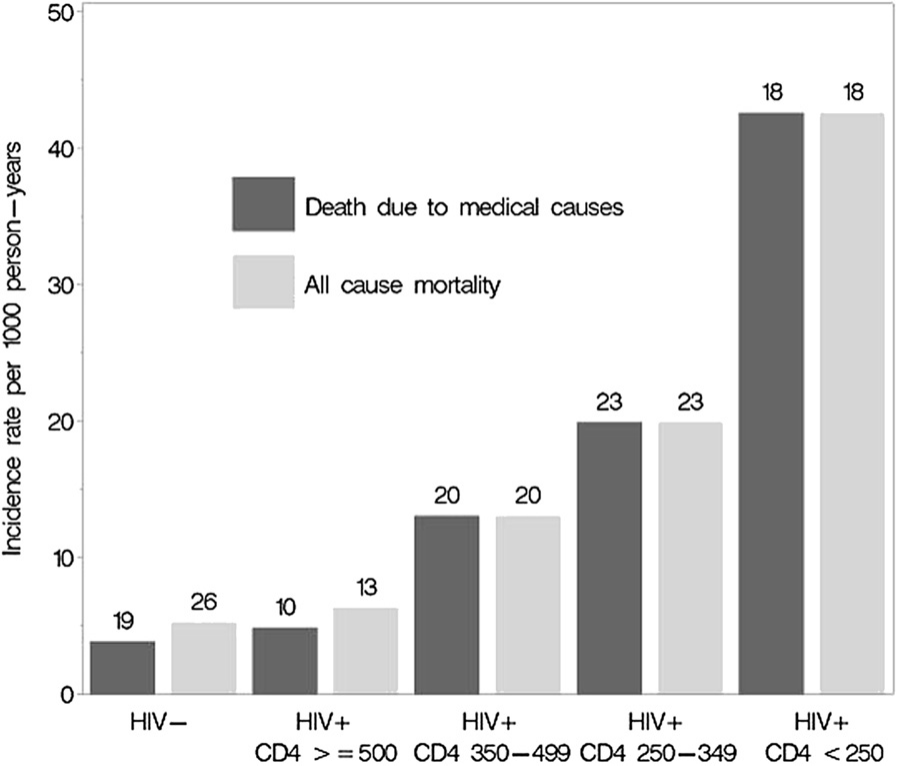
**Incidence of death by HIV-1 status and CD4 category for HIV-1-infected partners.** Numbers above bars indicate the absolute number of deaths that occurred in each category

Relative to the HIV-uninfected partner, the HIV-infected partner had excess medical mortality of 10.2 deaths/1000 person-years (95%CI: 6.9 – 13.5). We calculated the excess medical mortality attributable to HIV-1 infection by categories of CD4 count and plasma HIV-1 RNA, after adjusting for ART use (Table 5). Relative to HIV-1 uninfected persons, HIV-1 infected persons with a CD4 count of 500 cells/μl or greater had similar mortality due to medical causes (4.8 vs. 3.6 deaths/1000 person-years) (Figure 2). HIV-1 infected persons with a CD4 count in the range 350 – 499 cells/μl had an excess of 8.9 deaths/1000 person-years (95%CI: 3.8 – 14.0) compared to HIV-1 uninfected persons. The excess deaths rose further to 15.2 deaths/1000 person-years (95%CI: 8.1 – 22.2) for those with a CD4 count of 250 – 349 cells/μl and to 29.3 deaths/1000 person-years (95%CI: 13.9 – 44.7) for those with a CD4 count below 250 cells/μl.

**Table 5:** Excess mortality according to CD4 cell count and plasma HIV-1 RNA category among HIV-1 infected participants, after adjusting for ART use

	**Incidence ofmedical death**	**Estimated excess mortality per 1000 person-years (95% CI)**	***P*-value**	**Number needed to treat per year (95% CI) to avert 1 death**
**HIV-1 uninfected**	3.6	Reference	-	Reference
**HIV-1 infected**	13.7	10.2 (6.9, 13.5)	<.001	
**CD4 cell count**				
≥500	4.8	1.2 (−1.8, 4.2)	.44	851
350-499	13.1	8.9 (3.8, 14.0)	<.001	113
250-349	19.9	15.2 (8.1, 22.2)	<.001	66
<250	42.7	29.3 (13.9, 44.7)	<.001	34
**HIV-1 RNA**				
HIV-1 <10 K	7.7	2.0 (−1.1, 5.0)	.21	509
10 K-50 K	3.7	−0.6 (−3.4, 2.3)	.69	NA
50 K-100 K	13.9	6.7 (−0.6, 14.0)	.071	149
>100 K	46.9	43.0^†^ (29.6, 56.4)	<.001	23

† An interaction term was also present in the model between high HIV viral load and use of ART (p = .002). The excess mortality in the last row applies to those not on ART. For those on ART, mortality was not found to exceed that of HIV-1 uninfected (p = 0.58).

Furthermore, persons with a high plasma HIV-1 RNA experienced excess deaths in comparison to HIV-1 uninfected persons, with 43.0 excess deaths/1000 person-years (95%CI: 29.6 – 56.4) for HIV-1 infected persons with viral load greater than 100,000 (among those not taking ART). An interaction term between ART and high HIV viral load (>100 K) was present in the model. No excess mortality was found to be associated with plasma HIV-1 RNA prior to initiating ART for those who began ART (p = 0.58).

### Number needed to treat

Assuming that ART can effectively reverse immunosuppression and result in a life expectancy similar to that of HIV-1 uninfected persons, as has been observed in resource-rich countries that have ART initiation guidelines with higher CD4 counts, we calculated the number of HIV-1infected persons needed to treat with ART per year to avert a death at each stratum of CD4 cell count and HIV-1 viral load. At CD4 counts less than 250 cells/μl, 34 persons need to receive ART per year to avert one death attributable to HIV infection in this cohort with a median follow-up of 20 months. At CD4 counts in the range 250 – 349 cells/μl and 350 – 499 cells/μl, 66 and 113 persons, respectively, need to be treated to avert one death.

## Discussion

Our study shows that among African HIV-1 serodiscordant couples, who likely share many determinants of health such as socioeconomic status, nutrition and infectious disease exposures, mortality was higher in the HIV-1 infected partner at all but the highest levels of CD4 cell count (>500 cells/μl). We calculated the excess mortality within CD4 cell categories and found that appreciable excess mortality remains, even above CD4 cell counts that prompt initiation of ART according to current WHO guidelines (CD4 ≤ 350 cells/μl), or as currently implemented in many African countries (CD4 ≤ 250 or 200 cells/μl). Given that the excess mortality attributable to HIV-1 is noted at all but the highest levels of CD4 count, aligning the goal of reducing HIV-1-related mortality to revisions of treatment guidelines will likely require further increases in the CD4 threshold for ART initiation.

Few studies have examined the impact of HIV-1 infection with CD4 counts above 200 cells/μl in sub-Saharan Africa. A recent multicohort analysis examining mortality according to CD4 count at which ART was initiated did not include cohorts from Africa [8]. Analyses from the International epidemiological Databases to Evaluate (IeDEA) collaboration examining cohort data from West Africa and Southern Africa indicate that persons initiating ART with CD4 counts above 200 cells/μl have a residual excess mortality of 10.0 (for persons with less advanced clinical staging) to 35.9 deaths per 1000 person-years (for those with more advanced clinical disease) up to 24 months on therapy, compared to country-level estimates of HIV-free mortality [9]. The ART-LINC collaboration reported that mortality in the first year of ART was 6.4% in programs with active retention strategies, with mortality in the period immediately following initiation of therapy being higher than after six months [23]. However, only a small minority of subjects in that analysis were enrolled with a baseline CD4 count above 350 cells/μl, and those that were initiated therapy due to clinical progression or other conditions indicating advanced immunosuppression.

In our analysis, ART use was associated with a significantly increased risk of death. It is likely that this reflects confounding by indication [24], as ART is initiated in patients with the highest risk of mortality due to advanced HIV-1 disease. Further, since ART was prescribed outside of the study and there was a time lag between laboratory measures performed at study clinics and drug prescription, those on ART are likely to be at lower CD4 count than assessed in clinic; therefore, the observed associations between mortality and CD4 count are also likely to be conservatively biased. We observed that the risk of death fell after six months of therapy. In patients with low CD4 count, initiation of ART is associated with a substantial mortality from immune reconstitution inflammatory syndrome, which may have contributed to this finding [25]. Even though the final model included a number of factors that serve as indicators for ART initiation, we retained ART in the final model to fully describe factors associated with mortality.

In our study, both low CD4 count and high plasma HIV RNA level were independently predictive of poorer survival. This mirrors other studies from developed countries demonstrating an additional predictive role for measure of viremia [26,27]. Translation of this finding to a useful clinical stratification tool has been more complex. In African treatment programs, treatment initiation is guided by clinical findings and CD4 count, with rare or no measurement of viral load prior to ART. A clinical trial evaluating a combination HIV prevention strategy in Botswana will use viral load as an eligibility criterion [28] (NCT01583439).

Our findings add to the prior evidence of the prognostic significance of increased HIV-1 RNA levels for a given CD4 count. The association of level of viremia with mortality would also provide support for those advocating for increased use of viral load monitoring, while recognizing the success of African treatment programs has largely rested on their public health orientation, and that cost is a major factor for limited use. An additional consideration supporting wider use of viral load monitoring is that plasma HIV-1 RNA also predicts risk of transmission, and thus remains an important measure of the likelihood of further transmission of HIV-1 in the community [29,30].

Our study has several strengths. The observations are drawn from a large cohort of HIV-1 serodiscordant couples in Africa. Active outreach efforts to track those with loss to follow-up resulted in excellent retention, and it is likely that few or no deaths were not identified or reported. The diversity of 14 rural and urban sites in seven East and Southern African countries included participants living in a broader range of socioeconomic conditions than might be seen in highly localized study populations, such as demographic surveillance sites. The paired nature of the data provided a comparison group that can more carefully account for many of the socio-economic determinants of health than may be possible through a general community or country-level comparison.

We assumed that economic resources and access to care were shared equally within the couple. Such an assumption may not be correct in all cases, as the man may control a disproportionate amount of resources available within the family. Women have a marginalized status in some African societies [31]. Should such marginalization undermine our assumption of equitable access to resources and limit access to care, we might have expected to observe higher mortality among women participating in the trial. Our findings suggest otherwise, in that we observed a higher rate of all-cause death among men, once stratified by HIV-status, albeit not a statistically significant finding. Thus we found no reason to infer that power differentials related to gender were significantly impeding women’s access to care, or if present, were overshadowed by differential healthcare seeking behaviour [32,33]. Furthermore, the couples that chose to participate in a discordant couples’ trial may not reflect couples in a general population. The increased mortality among men has been noted in HIV-1/TB co-infected patients attending care in Soweto [34]. Other studies have also noted higher mortality among men related to trauma [10]. There is a need to investigate barriers to men accessing care and understand factors associated with higher mortality in men.

HIV-1 is also known to have deleterious impacts on the socioeconomic status of households [35]. It is not clear what effect, if any, such a phenomenon might have had in this cohort, and whether any negative impacts would have more significantly impacted the HIV-1 infected or HIV-1 uninfected participant. Most sites conducted the study in or adjacent to a health care facility, which may have also skewed the distribution of socio-economic status of study participants. Differences in socio-economic status between facility-based and community-based patient samples attending HIV-1 care have been noted in the African context [36].

## Conclusions

In summary, our data confirm the large contribution from HIV-1 to mortality in adults in the African setting. Our data also indicate that the excess mortality was highest in those with CD4 counts below 250 cells/μl, although a significant excess remained at all CD4 categories below 500 cells/μl. Thus, our results support increasing the CD4 threshold for treatment initiation, together with expanding HIV testing for asymptomatic persons in the community, if the goal of reducing HIV-1-related excess mortality is to be achieved. Determination of plasma HIV-1 RNA measures may further aid in identifying those individuals at higher risk of rapid disease progression and HIV-1 transmission.

## Competing interest

All authors report no conflicts of interest

## Authors’ contributions

GdB, AM, AW designed the study and AM did the analysis. All authors contributed to data collection and writing of the report and all approved the final draft. GdB, AM and AW wrote the initial draft and vouch for the data, analysis, interpretation, and manuscript submission.

## Funding

Bill and Melinda Gates Foundation (grant ID #26469); NIH grant AI-30731 (AM, AW) and AI-083034 (JMB, CC).

## Pre-publication history

The pre-publication history for this paper can be accessed here:

http://www.biomedcentral.com/1471-2334/12/277/prepub

## Supplementary Material

Additional file 1 Methods. Click here for file
