# Supplementary material for: Mortality in members of HIV-1 serodiscordant couples in Africa and implications for antiretroviral therapy initiation: Results of analyses from a multicenter randomized trial
Source: BMC Infect Dis. 2012 Oct 30;12:277. doi: 10.1186/1471-2334-12-277 (PMC3582549; doi:10.1186/1471-2334-12-277)

# SUGGESTED APPENDIX

To compute confidence intervals for excess mortality from Poisson regression mortality rates, we derived variance estimates using the delta-method.

As an example, let HIVpos be an indicator for HIV positivity (1=yes, 0=no) and HVL be an indicator for high HIV viral load (1=high HIV viral load, 0=either low HIV viral load or HIV negative).

Then the Poisson regression equation for mortality can be written as:


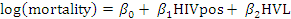


And it can be exponentiated to be place on the scale of morality rate.


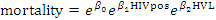


Define a new function
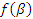
 as the excess mortality, or different in mortality, due to being HIV seropositive *and* having high HIV viral load, relative to HIV seronegatives.

Excess morality is:


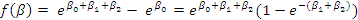


We compute the variance of the excess mortality measure
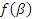
as
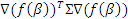
, where


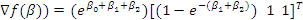
 and


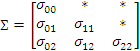
 is the variance-covariance matrix of the β vector.

The variance in excess mortality is:


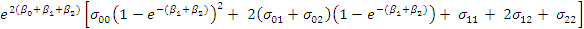

Supplement: Additional file 1 — Methods. [file 1471-2334-12-277-S1.doc]
